# Species evolution: cryptic species and phenotypic noise with a particular focus on fungal systematics

**DOI:** 10.3389/fcimb.2025.1497085

**Published:** 2025-02-04

**Authors:** Anusha H. Ekanayaka, Samantha C. Karunarathna, Saowaluck Tibpromma, Arun Kumar Dutta, Danushka Sandaruwan Tennakoon, Anuruddha Karunarathna, Ekachai Chukeatirote, Dong-Qin Dai, Steven L. Stephenson, Sajeewa S. N. Maharachchikumbura, Chao Liu, Alan J. L. Phillips

**Affiliations:** ^1^ Center for Yunnan Plateau Biological Resources Protection and Utilization, College of Biology and Food Engineering, Qujing Normal University, Qujing, China; ^2^ Department of Urban Bioresources, Faculty of Urban and Aquatic Bioresources, University of Sri Jayewardenepura, Nugegoda, Sri Lanka; ^3^ National Institute of Fundamental Studies (NIFS), Kandy, Sri Lanka; ^4^ Molecular & Applied Mycology Laboratory, Department of Botany, Gauhati University, Guwahati, Assam, India; ^5^ Guangdong Provincial Key Laboratory for Plant Epigenetics, Shenzhen Key Laboratory of Microbial Genetic Engineering, College of Life Science and Oceanography, Shenzhen University, Shenzhen, China; ^6^ Department of Entomology and Plant Pathology, Faculty of Agriculture, Chiang Mai University, Chiang Mai, Thailand; ^7^ School of Science, Mae Fah Luang University, Chiang Rai, Thailand; ^8^ Department of Biological Sciences, University of Arkansas, Fayetteville, AR, United States; ^9^ School of Life Science and Technology, Center for Informational Biology, University of Electronic Science and Technology of China, Chengdu, China; ^10^ Universidade de Lisboa, Faculdade de Ciências, Biosystems and Integrative Sciences Institute (BioISI), Lisbon, Portugal

**Keywords:** biodiversity, ecology, evolution, phylogeny, taxonomy

## Abstract

The evolution of a species can be understood in the context of two major concepts—the cryptic species concept and the phenotypic noise concept. The former represents morphologically indistinguishable but genetically distinct evolutionary lineages, while the latter represents the phenotypic variations of an isogenic population. Although the concept of cryptic species currently represents a general topic, its effect on other aspects of biology, such as biodiversity, ecology, evolutionary biology, and taxonomy, is still unclear. In particular, cryptic species cause complications and prevent the development of a clear taxonomy. The phenotypic noise concept or phenotypic plasticity generally refers to the various expressions of phenotypes in different environments. Hence, the cryptic species concept refers to genetic variations, while the phenotypic noises concept is about non-genetic variations. Although both concepts are opposites, they each contribute significantly to the evolutionary process of an organism. Despite the extensive research studies and publications discussing those two concepts in separate accounts, a concise account that combines and compares both concepts are generally lacking. Nevertheless, these are essential to understand the evolutionary process clearly. This review addresses the available literature on this topic, intending to provide a general and overall discussion on both the cryptic species concept and the phenotypic noise concept and their effect on evolution, ecology, biodiversity, and taxonomy with a special focus on fungal systematics. hence, several fungal case studies representing the two concepts are presented, compared, and discussed for a better understanding.

## 1 Introduction

Evolution occurs when a group of organisms undergoes gradual changes in their properties over generations. The changes in genome, physiology, or morphology make the particular group of organisms in a population different from their recent ancestors, causing those individuals in question To achieve reproductive isolation from other individuals within the population who share a particular ecological niche, maintaining the ability to interbreed (Mayr, 1982; Aldhebiani, 2018). This process is referred to as speciation. This evolutionary process or speciation event results from a complex series of circumstances that directly connect with the species concept. The species circumscription and delimitation are based on two major concepts—the cryptic species concept and the phenotypic noise concept.

The matter of delineating species has been compounded by a fundamental challenge related to what constitutes a species. This challenge arises from divergent perspectives among biologists regarding the definition of species. Over the past fifty years or so, various factions within the biological community have advocated for distinct and sometimes conflicting species concepts, as documented by (Mayden, 1997; De Queiroz, 1998; Harrison, 1998). Among them, Mayden (1997) enumerated 24 distinct species concepts with numerous additional alternative definitions. A definition here refers to a concise depiction of a concept, meaning that a single species concept may be associated with multiple definitions that vary in minor linguistic nuances. Many of these concepts and their corresponding definitions are incompatible, often leading to disparate conclusions regarding the delineation and enumeration of species. Consequently, the conundrum of species concepts, which is related to the discord of the current theoretical concept of species, is intricately bounded by the challenge of determining species’ boundaries and quantities based on empirical evidence (De Queiroz, 2007).

More commonly discussed species concepts include morphological, biological, evolutionary, and phylogenetic concepts (Taylor et al., 2000). Among them, the biological and evolutionary species concepts are widely used. Currently, there are conflicts among these terms. A biological species refers to inter-fertile populations reproductively isolated from other such groups and occupying a distinct ecological niche (Mayr, 1982). An evolutionary species refers to a single lineage of ancestor-descendant populations with a unique evolutionary history and the ability to maintain its identity from other such lineages. It also fits into its ecological niche (Simpson, 1961; Wiley, 1981; Paris et al., 1989). However, there is a congruence between the theoretical and operational species concepts. The operational species concept surpasses the theoretical one in prevalence, which causes conflicts in other related areas of evolution, ecology, biodiversity, and taxonomy (Mayden, 1997; Taylor et al., 2000). Hence, a better understanding of the causes for the congruence of the theoretical and operational species concepts is essential.

As the evolutionary species concept defines a species as a single lineage of ancestor-descendent populations, the theoretical species concept was primarily considered the evolutionary species concept (Taylor et al., 2000). However, current understandings of evolutionary consequences deviate from this primary idea about the theoretical species concept. With the slow process of evolution, specific individuals of a biological species acquire minor changes in their genotype. At a specific stage of this evolution, the newly changed individuals become non-fertile with the original population, and they thus represent a distinct evolutionary lineage (Paris et al., 1989; Struck and Cerca De Oliveira, 2019). However, both groups (original and newly changed) can still be morphologically indistinguishable (Struck and Cerca De Oliveira, 2019). This is the stage at which a cryptic species arises. It is supposed that as time passes, those two populations diverge gradually in their morphology and physiology to be suited to unique ecological conditions (Wiley, 1981; Paris et al., 1989; Struck and Cerca De Oliveira, 2019). Although most authors adopt the evolutionary species concept, they often recognize and classify species based only on distinctive phenotypic characteristics. Afterward, the classification followed also becomes morphologically based, the basis upon which the taxa can be easily identified. Generally, the evolutionary or phylogenetic species concept is superior to morphology-based and biological species concepts. Therefore, the morphological species concept is neither an evolutionary nor a biological species concept. As a result, a conflict arises between theory and practice (Paris et al., 1989; Struck and Cerca De Oliveira, 2019). Despite everything, the morphological species concept has become a more significant inference to the biological or evolutionary species concepts and the foundation for the cryptic species concept (Dobzhansky, 1982; Aldhebiani, 2018; Struck and Cerca De Oliveira, 2019). On the other hand, the morphological species concept is strongly influenced by ecological and environmental conditions. Therefore, gene expression can vary with the environment and is reflected in phenotype variations. Most importantly, even if the phenotype changes, the genotype is still the same (West-Eberhard, 2008; Levin, 2013; de Vienne, 2021).

The cryptic species concept also called the sibling species concept, describes two or several species that are indistinguishable in their phenotypes but differ in their genotypes (Zúniga-Reinoso and Benítez, 2015; Korshunova et al., 2019). Hence, in a morphological concept, they have been classified as a single nominal species (Zúniga-Reinoso and Benítez, 2015). Phenotypes of organisms within an isogenic population are not identical since they exhibit some degree of difference/variation from each other (Kaneko and Furusawa, 2008). Phenotypic noise refers to these variations in a phenotype developed by an individual genotype; it is also referred to as phenotypic plasticity (West-Eberhard, 2008). The environment and history of an organism influence the development of the variability in the cellular phenotype while the genotype is still unchanged (Raser and O’shea, 2005). As such, a single species can be given different names simply because of its phenotypic plasticity in different habitats. Both concepts are contradictory to one another and have created confusion in modern taxonomy. Both have significantly influenced other related research areas, including evolutionary biology, ecology, and biodiversity. Since morphology is a contentious marker in taxonomy, it hides cryptic species that share certain phenotypic characteristics but are otherwise distinct. As a result of the presence of cryptic species or the inability to recognize existent species, actual biodiversity levels are underestimated or overestimated (Lefébure et al., 2006; Padial et al., 2010).

Although cryptic species are more or less morphologically indistinguishable, their genotypes are significantly different (Wei et al., 2021). Therefore, molecular phylogenetic tools are the only fast, reliable, and efficient methods to discover species and face the taxonomic problems caused by cryptic species (Jörger and Schrödl, 2013; Korshunova et al., 2019). This is again applicable to the phenotypic noise concept. Molecular phylogenetic tools clearly recognize the different phenotypes influenced by the environment of the same species.

The concept of cryptic species dates back about three centuries, and the first record was provided by Derham in 1718 (*in* Ray and Willughby, 1718). Later authors have defined cryptic species in different ways. The first explicit definition for cryptic species was “population systems which were believed to belong to the same species until genetic evidence shows the existence of isolating mechanisms separating them” (Stebbins, 1950). Korshunova et al. (2019) recently defined cryptic species as “species which manifest low morphological but considerable genetic disparity”. Considering several definitions for cryptic species (Stebbins, 1950; Paris et al., 1989; Zúniga-Reinoso and Benítez, 2015; Korshunova et al., 2019; Struck and Cerca De Oliveira, 2019), they are considered to represent distinct evolutionary lineages and are reproductively isolated; however, morphologically indistinguishable, they have historically been misinterpreted as members of a single species.

The discussions on phenotypic noise began with the origin of the term “phenotype”. The first clear definition of phenotype was “All types of organisms, distinguishable by direct inspection or only by finer methods of measuring or description” (Johannsen, 1909). The general consideration among scientists is that the phenotype refers to the observable characteristics in an individual and reflects the genotype of an organism resulting from the expression of genes. The concept of phenotypic noise describes how the environment exerts an effect on gene expression and then how phenotypes vary based on environmental conditions (West-Eberhard, 2008; Levin, 2013). Phenotypic noise is also defined as the environmental sensitivity of a genotype (West-Eberhard, 2008).

One of the earliest examples of cryptic species is the genus *Drosophila*, which includes fruit flies (Dobzhansky and Epling, 1944; Dobzhansky, 1951). The two species *D. pseudoobscura* and *D. persimilis* were originally treated as one species. Later, in laboratory cultures, researchers observed these two species were reproductively isolated. Further studies identified and confirmed their differences in morphology (in wings and male genitalia), physiology, behavior, and chromosome morphology (Dobzhansky and Epling, 1944; Dobzhansky, 1951). Another example of cryptic species is provided by Adams et al. (2014), who explored the hyper-cryptic species complexes of Australian freshwater fish collections based on allozyme, mtDNA, and morphological data. They found that these species, across their broad geographic range, revealed a 1500% increase in species-level biodiversity, including 15 distinct species of phenotypically similar minnows (*Phoxinus* spp, a small freshwater fish) from different lakes (Adams et al., 2014). Investigations of *Phoxinus* communities of adjacent freshwater ecosystems of the westernmost part of the Po River basin in Italy, based on mitochondrial cytochrome oxidase I (COI) sequences from 239 specimens, revealed a complex of species of *Phoxinus*, consisting of *P. septimaniae*, *P. csikii*. and *P. lmaireul*, which are morphologically indistinct (De Santis et al., 2021). In another example, phylogenetic analyses of 37 samples from 13 taxa of the Asian forked fern genus (*Dicranopteris*) from five chloroplast gene regions (rbcL, atpB, rps4, matK, and trnL-trnF) showed that *D. linearis* is polyphyletic, which suggests that there is cryptic diversity within the species. Further comparisons erected the new species *Dicranopteris austrosinensis* and *D. baliensis* (Wei et al., 2021). Peintner et al. (2019) identified and analyzed *Fomes* strains from different habitats in Italy and Austria using rDNA ITS region phylogenetics. These findings proved the existence of subclades within the *Fomes fomentarius* clade and led to the formal recognition of the new species *Fomes inzengae* (Peintner et al., 2019). Researchers analyzed 96 Japanese specimens of the *Hypholoma fasciculare* complex, a group of common wood-decomposing fungi, using mitochondrial ribosomal RNA (mt-rRNA) sequences, nuclear ITS region, and 24 single-copy genes. The results showed that the *H. fasciculare* complex encompassed two species, *H. fasciculare* and *H. subviride* (Sato et al., 2020).

The phenotypic noise concept has been discussed at length in several research papers (Raser and O’shea, 2005; West-Eberhard, 2008; Levin, 2013; de Vienne, 2021). Some interesting studies on phenotypic noise include the identification of intraspecific phenotypic variation and morphological divergence of *Folsomia candida* by Tully and Potapov (2015) and phenotype microarray profiling of *Staphylococcus aureus* by von Eiff et al. (2006). Compared with the cryptic species concept, the phenotypic noise concept lacks detailed studies. However, many confusing points remain on these topics. Moreover, the combined effects of the cryptic species concept and the phenotypic noise concept on other fields, such as taxonomy, evolution, ecology, and biodiversity require further studies. Hence, the present study reviews and discusses the influence of the cryptic species concept and the phenotypic noise concept on taxonomy, evolution, ecology, and biodiversity research. Future research trends related to these concepts are summarized and discussed.

Further, fungi are versatile and reflect their diversity in morphology, ecology, physiology, and phylogeny. Fungi rank as the second most diverse group of organisms in terms of species, followed by insects (Purvis and Hector, 2000; Antonelli, 2023). Among fungi, a remarkable range of morphological diversity exists, spanning from single-celled yeasts to substantial ‘fruiting bodies’ that can generate trillions of spores (Stajich et al., 2009). Numerous fungi’s developmental phases occur within or on intricate substrates like soil, wood, plants, or animals, rendering them challenging to observe. Consequently, developmental stages and true ecological diversity of most fungi remain undiscovered (Rodriguez et al., 2004). Both fungi that produce large reproductive structures (e.g., mushrooms and truffles) and those that make no reproductive structures apart from the meiocytes themselves (e.g., yeasts) show such complex reproductive models (Stajich et al., 2010). The most intricate formations within the fungal realm are the multicellular sexual fruiting bodies, characterized by well-defined fungal tissues and various cell types (Kües et al., 2018). Nevertheless, unicellular yeasts show huge complexities in their life cycles (Yurkov et al., 2015). They also produce numerous bioactive molecules, which are helpful for various research areas such as agriculture, industrial, and pharmaceuticals (Raja et al., 2017). The estimated number of fungal species on Earth was approximately 12 million (ranging from 11.7 to 13.2 million), a significant increase from the previous estimate of 2.2 to 3.8 million species obtained through various estimation techniques. A team of fungal experts recently assessed the fungal diversity in the world using four main academic pathways viz. scaling laws; fungus: plant ratios; actual versus previously known number of species; and DNA-based studies; according to them, there are likely to be 2–3 million species of fungi, with a best estimate of 2.5 million (Antonelli, 2023). On the other hand, molecular phylogeny has revealed the existence of numerous cryptic species within this diverse fungal kingdom (Wu et al., 2019). However, recent studies revealed that fungal species circumscription and delimitation are a considerable challenge (Raja et al., 2017; Baturo-Ciesniewska et al., 2020; Stengel et al., 2022). Specifically, fungal species delimitation is complicated by cryptic species (Crespo and Lumbsch, 2010; Shivas and Cai, 2012; Peintner et al., 2019; Sato et al., 2020; Wang et al., 2022). Phenotypic noises or plasticity also cause controversies incorrect species identification in fungi (Lehner, 2010; Behm and Kiers, 2014). Hence, this review is also expected to improve the identification of fungal species and aid in developing future fungal research applications.

## 2 Application of the concepts in fungal systems

To review the available literature related to cryptic species and phenotypic noises concepts, various sources such as scholarly papers, digital databases, and personal communications were used. Data on the cryptic species concept and the phenotypic noise concept were collected primarily from “Google Scholar,” “ResearchGate,” “PubMed,” and “Web of Science.” The keywords such as species concept, cryptic species, phenotypic noises, biological, phenotype, genotype, ecology, biodiversity, and evolution were used in different combinations to derive references for the review. The derived literature was reviewed and analyzed to understand the fundamentals of cryptic species and phenotypic noise concepts and an in-depth literature review was done to determine their effect on biodiversity, ecology, evolutionary biology, and taxonomy.

Several suitable examples to reflect the occurrence of cryptic species and phenotypic noises within species circumscription and delimitation, were obtained from recently published literature with the approval of the original authors (Thambugala et al., 2015; De Silva et al., 2020). Authors re-examined a few specimens to confirm their morphological characteristics *viz. Marasmius imitarius* specimen was loaned from the Calcutta University herbarium (CUH), *Rhytidhysteron neorufulum* specimens were obtained from the Mae Fah Luang University herbarium (MFLU), and *Misturatosphaeria aurantiacinotata* from the National Museums of Kenya/East African Herbarium (EA). The specimens of *Rhytidhysteron neorufulum* and *Misturatosphaeria aurantiacinotata* were initially examined with a Motic SMZ 168 stereomicroscope (Motic Asia, Kowloon, Hong Kong), and further observations were made with a Nikon ECLIPSE 80i compound microscope (Nikon Instruments Inc., Melville, New York, USA) with photographs recorded with a Canon 550D digital camera (Canon Inc., Ota, Tokyo, Japan). Hand-cut sections were mounted in sterile water for study and photographs. Tarosoft (R) Image Frame Work programme (Version: 1.3) was used to make measurements, and Adobe Photoshop CS3 extended version 10.0 was used to process the images used for the figures (Adobe Systems, USA). The specimen of *Marasmius imitarius* was studied with a Dewinter ‘crown’ trinocular microscope (Dewinter Optical Inc., New Delhi). Photographs were taken with the dedicated camera attached to the microscope.

The phylogenetic trees were reproduced using published sequence data in the NCBI database related to above mentioned examples. For each gene, sequences were aligned using MAFFT v. 7 (http://mafft.cbrc.jp/alignment/server/index.html) and manually adjusted in BioEdit v. 7.0.4 (Hall, 2004) where necessary. Datasets were concatenated with FaBox (1.41) (Villesen, 2007). Ambiguously aligned regions were excluded and gaps were treated as missing data.

As a model of evolution, the GTR+G+I substitution model was used. Maximum likelihood (ML) phylogenetic analyses were conducted using the RAxML-HPC2 Workflow on XSEDE (8.2.9) available through the CIPRES web portal (Miller et al., 2011; Stamatakis, 2014). Each ML tree bootstrap analysis was run using 1000 in-depth replicates with identical parameters. Markov chain Monte Carlo (MCMC) sampling in the CIPRES web portal (Miller et al., 2011) with MrBayes on XSEDE was used to calculate posterior probabilities (PP). Four Markov chains were run in parallel for two million generations, with tree samples taken every thousand generations. The temperature parameter of the MCMC heated chain was fixed at 0.2. Tracer v.1.5 (Rambaut and Drummond, 2009) was used to analyze the log-likelihood score distribution and determine whether more search iterations were necessary to reach convergence. Topologies below the asymptote (the top 80% of the sample) were rejected as part of the burn-in phase, and the remaining trees were used to determine posterior probabilities in the majority rule consensus tree.

### 2.1 Cryptic species examples

#### 2.1.1 Example - *Marasmius imitarius*

The genus *Marasmius* is cosmopolitan in distribution and can be recognized as those taxa that possess reviving basidiomata with a sulcate or corrugated pileus, a centrally placed cartilaginous stipe, and white basidiospores (Fries, 1835). *Marasmius imitarius* is morphologically very similar to many other species in the same genus (Wannathes et al., 2009). In fact, the specific epithet suggests how phenotypically this species resembles others in the genus. The taxon can be identified morphologically by its light brown sulcate pileus with a darker brown to reddish brown center, its distant (10–12), cream-colored lamellae with brown edges, its clavate basidiospores with a mean range of 17.8–19.2 × 4.1–4.7 μm, the absence of pleurocystidia and caulocystidia, and the fact that it grows primarily on woody twigs (Wannathes et al., 2009). According to Wannathes et al. (2009), most of these morphological features of *M*. *imitarius* overlap with other taxa like *M*. *bambusiniformis*, *M. mazatecus*, *M. striaepileus*, and *M. sierraleonis*.

**Figure 1:**
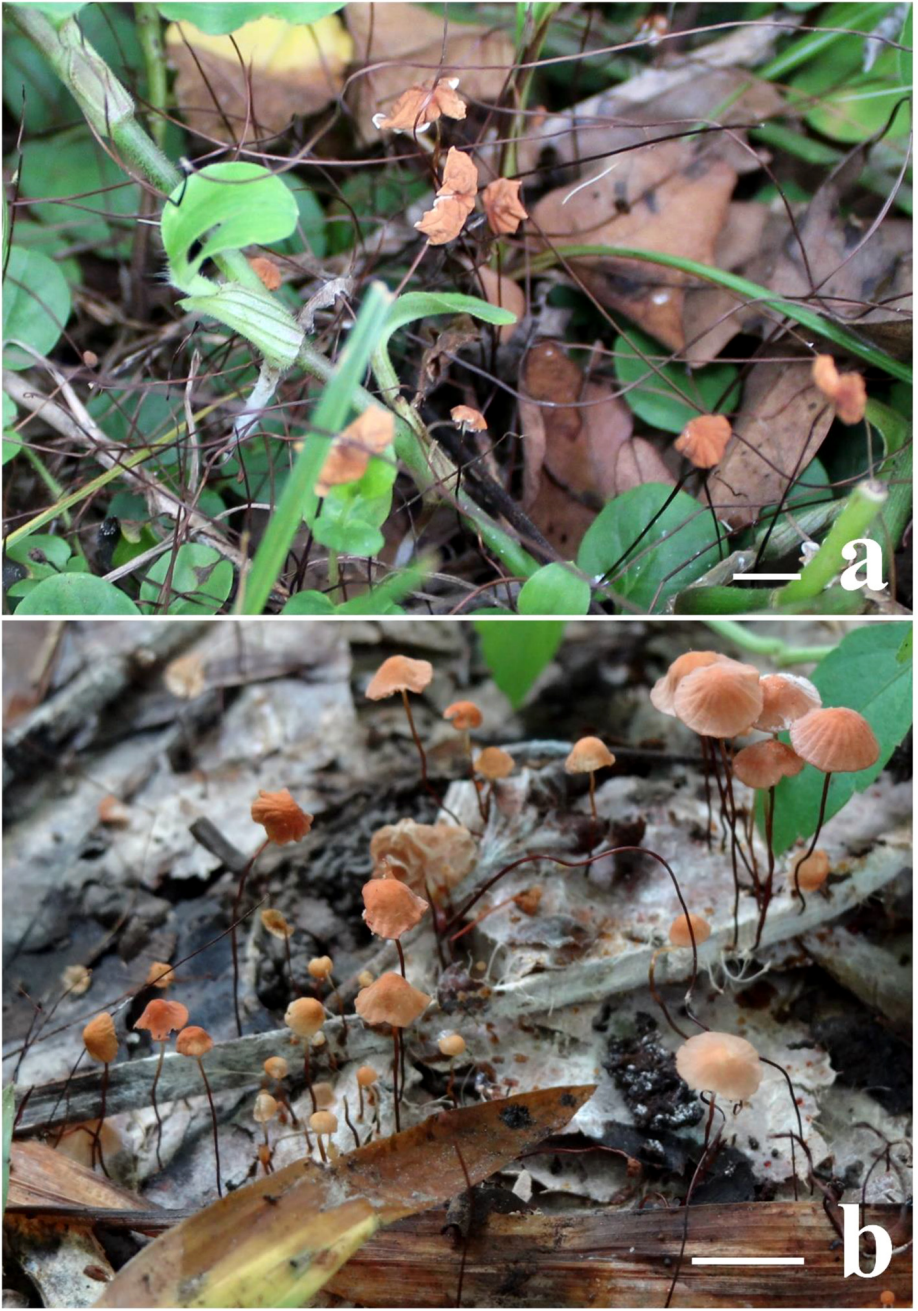
Field photographs of basidiomata. **(A)** *Marasmius imitarius* (CUH AM078), **(B)** *Marasmius bambusiniformis* (author’s (Arun Kumar Dutta) personal collections, AKD 382/2014). Scale bars = 10 mm.

To confirm the observation reported by Wannathes et al. (2009), we examined one of the Indian collections of *Marasmius imitarius* (CUH AM078) and *M*. *bambusiniformis* (AKD 382/2014, the author’s (Arun Kumar Dutta) personal collection; **Figure 1**). The examination revealed that the taxon *M*. *imitarius* (CUH AM078) possesses morphological characteristics such as a small (5–17 mm diam.), obtusely conical to convex pileus that matures to convex to plano-convex with a slight central depression, often with an upturned margin showing the faces of brownish orange to reddish orange lamellae with a smooth to minutely pruinose surface; distant (L = 9−11, l = 0−1) white to cream lamellae often with brown edges; fusoid to clavate basidiospores (14.5−21.5 × 3.5–6.8 µm); absence of pleurocystidia; *Siccus*-type cheilocystidia with clavate to pyriform main body (7.5−14.5 × 4−9 µm) and cylindrical apical setulae (3.5−7.5 µm); a hymeniform pileipellis consisting of *Siccus*-type broom cells with main body measuring 10−21.5 × 6.5−10 µm, and apical setulae measuring 3.0−7.0 µm; absence of caulocystidia; and the presence of clamp-connections in all tissues.

Further observation of *M*. *bambusiniformis* (collection no. AKD 382/2014) revealed that *M*. *bambusiniformis* consists of features such as a small (5–10 mm diam.), conic to convex or often applanate pileus, with smooth to slightly pruinose surface colored light brown to brownish yellow with brownish orange center; distant to subdistant (L = 18–21, l = 0–1) cream lamellae; fusoid to clavate basidiospores (14–19.5 × 3.5–6.0 µm); absence of pleurocystidia; *Siccus*-type cheilocystidia with clavate to pyriform main body (11.5–16 × 5–8 µm) and cylindrical apical setulae (3.5−8 µm); a hymeniform pileipellis consisting of *Siccus*-type broom cells with a main body measuring 12.5–20 × 7–8.5 µm, and apical setulae measuring 3.5−8.0 µm; absence of caulocystidia; and the presence of clamp-connections in all tissues.

**Figure 2:**
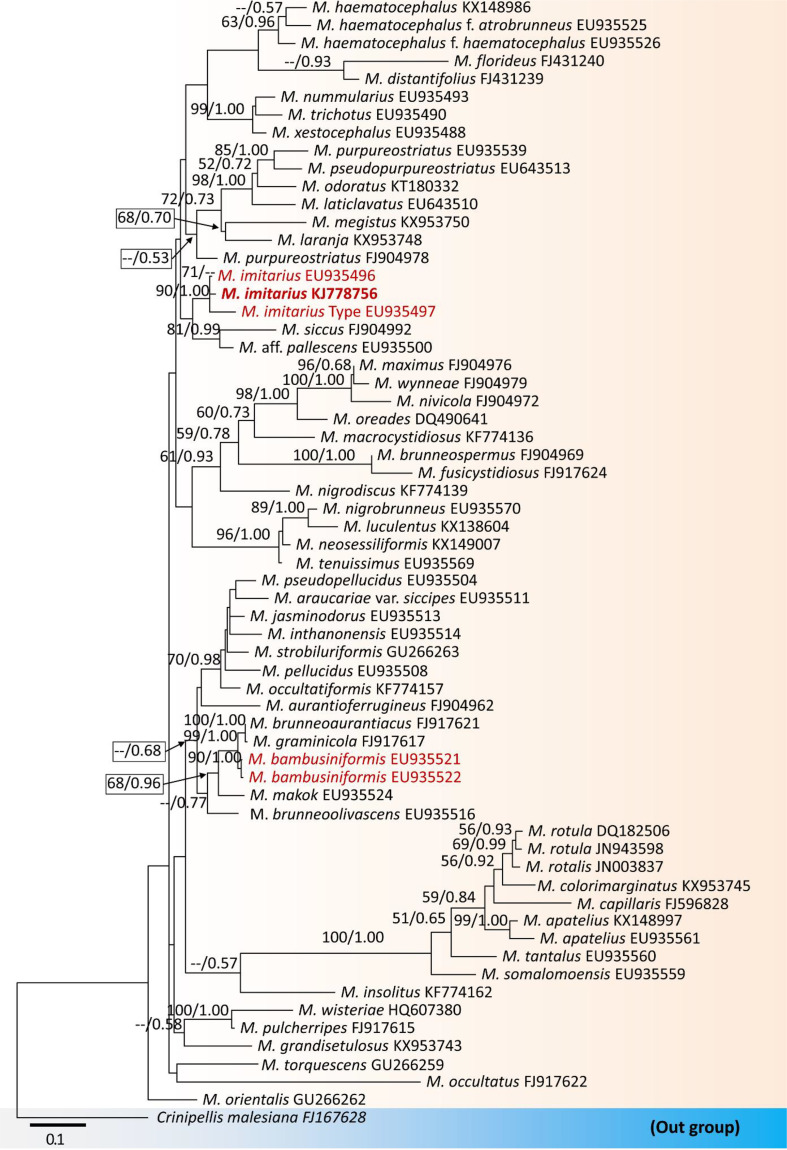
Maximum likelihood tree constructed using ITS rDNA sequence data. The tree is rooted with *Crinipellis malesiana*. Morphologically look-a-like taxa, *Marasmius imitarius* and *M. bambusiniformis* are in red. The collected specimen of *M*. *imitarius* is in bold. Bootstrap support values ≥50% from the maximum likelihood (ML) and Bayesian posterior probabilities (BYPP) values ≥0.50 are given above the nodes, respectively. The abbreviation ‘*M*.’ represents the genus *Marasmius*.

Hence, our study on the detailed morphology of both taxa (like *M*. *imitarius* and *M*. *bambusiniformis*) also suggests that differentiating these two taxa based solely on morphological features is insufficient. However, phylogenetic analyses based on the nrITS DNA data of this study (**Figure 2**) show *M. imitarius* lies far away from *M. bambusiniformis*, and previous studies also show similar results with this study (Wannathes et al., 2009). In fact, based on phylogenetic analyses, *M. imitarius* is not closely related to *M. bambusiniformis* (**Figure 2**).

### 2.2 Phenotypic noise concept examples

#### 2.2.1 Example 1- morphology variations of *Rhytidhysteron neorufulum*

**Figure 3:**
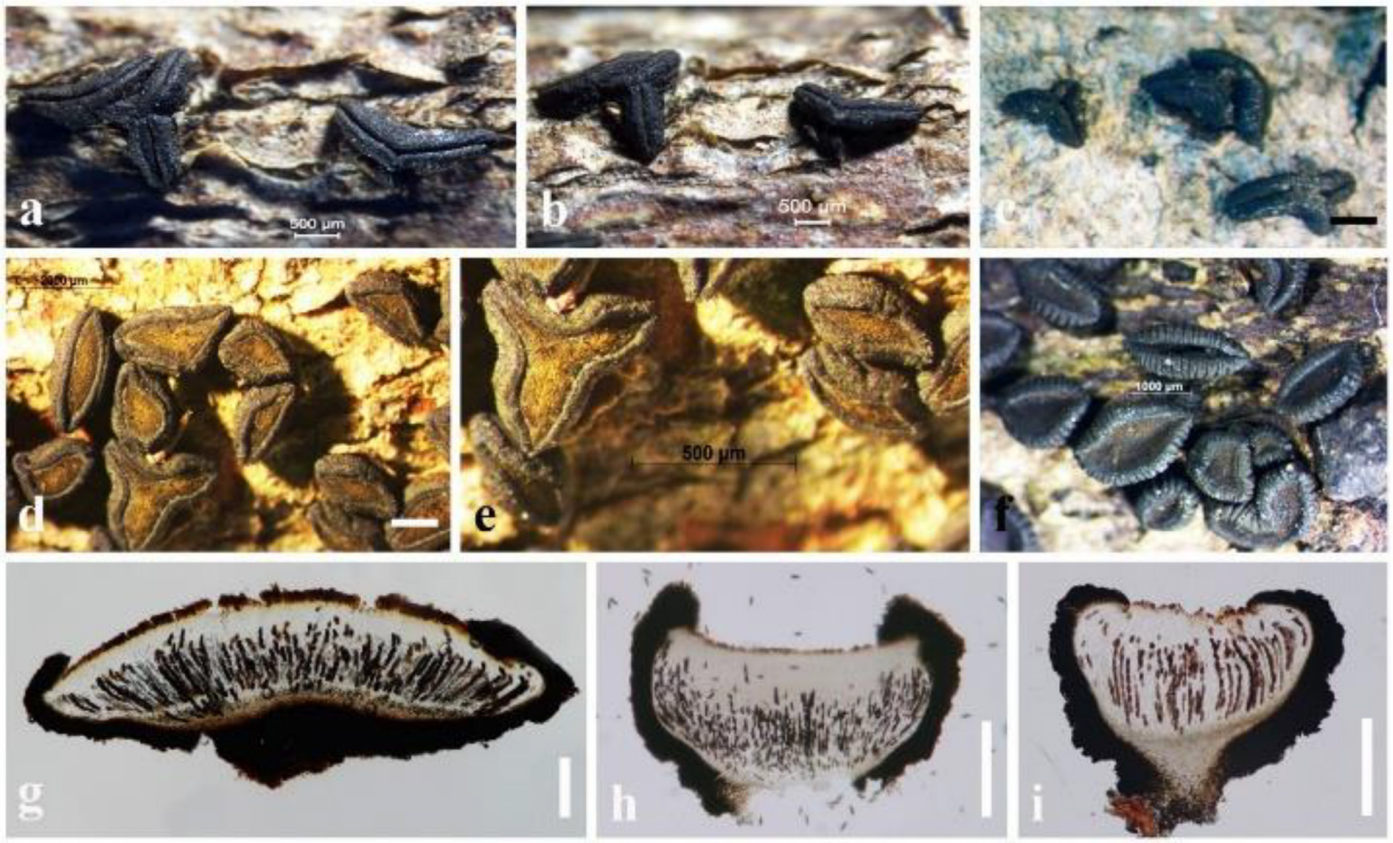
Ascomata variation in *Rhytidhysteron neorufulum*. **(A, B)** Ascomata of MFLU 18-2644. **(C)** Ascomata of MFLU 21-0248. **(D–F)** Ascomata of MFLU 14-0608. **(G)** Vertical section of MFLU 14-0608. **(H, I)** Vertical section of MFLU 18-2644. Scale bars: **(A–C, E)** = 500 μm, **(D)** = 2000 μm, **(F)** = 1000 μm, **(G–I)** = 300 μm.

The hysteriaceous genus *Rhytidisteron* belongs to the family Hysteriaceae in the Dothideomycetes (Thambugala et al., 2016). These fungi have a worldwide distribution mostly as saprobes, endophytes or weak pathogens on woody plants and rarely as human pathogens (Thambugala et al., 2016; De Silva et al., 2020; Cobos-Villagrán et al., 2021). Species of *Rhytidisteron* are characterized by having large, conspicuous, elongated, superficial, carbonaceous to coriaceous, navicular ascomata, mostly opening by a longitudinal slit and with thick-walled ascospores (Thambugala et al., 2016). Since these fungi express varied shapes of ascomata depending on the environmental conditions, their ascomata traits are quite fascinating. For instance, their ascomata are disc-shaped (irregular opening/apothecial-shape) under wet conditions, but when dry, they fold at the margin, forming an elongated slit (hysterothecial-shape) (Thambugala et al., 2016; Cobos-Villagrán et al., 2021). In some cases, the same specimen may also contain disc-shaped and hysterothecial-shaped ascomata. For example, we observed the ascoma characteristics of several isolates of *Rhytidhysteron neorufulum* (MFLU 18-2644, MFLU 21-0248 and MFLU 14-0608) and found that all consist of both disc-shaped and hysterothecial-shaped ascomata (**Figure 3**). Thus, their apothecial or hysterothecial behavior could not be considered as a valid trait for delimitation of species of *Rhytidisteron* as those differences can be attributed to environmental changes.

**Figure 4:**
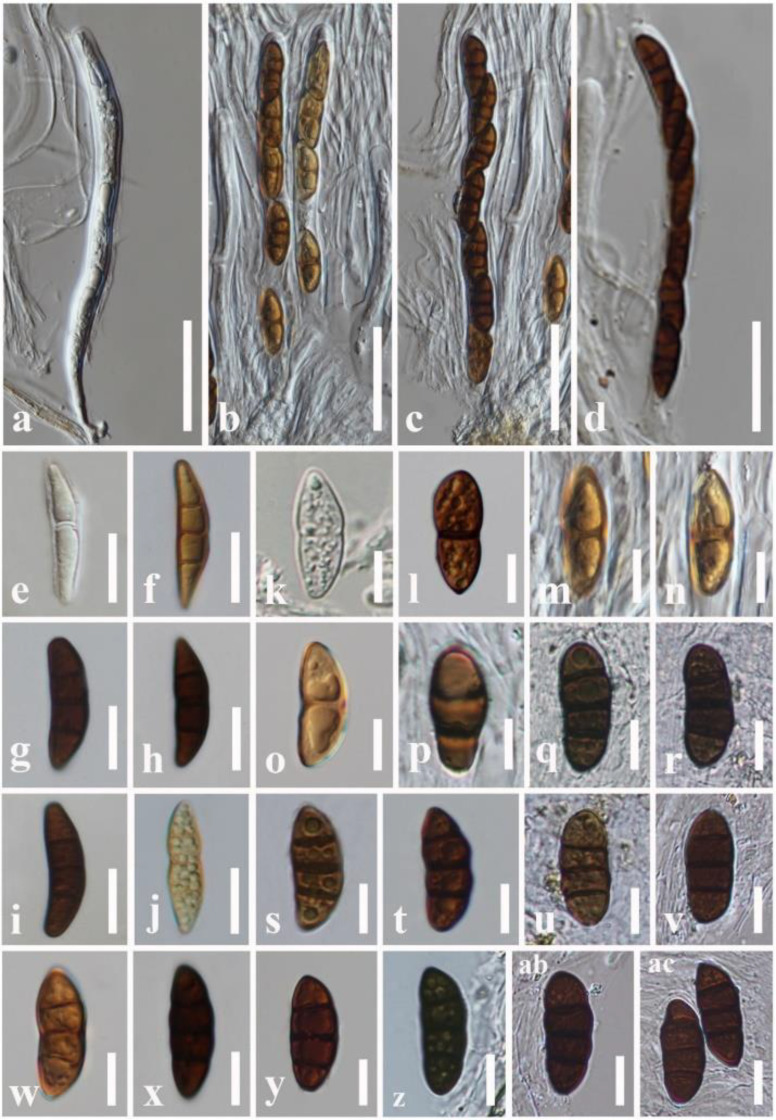
Variations in *Rhytidhysteron neorufulum* ascospores. **(A, B)** Asci of MFLU 18-2644. **(C, D)** Asci of MFLU 14-0608. **(E–J)** Ascospores of MFLU 14-0608. **(K–R)** Ascospores of MFLU 18-2644. **(S–AC)** Ascospores of MFLU 21-0248. Scale bars: **(A–D)** = 50 μm, **(E–J)** = 15 μm, **(K–AC)** = 12 μm.

**Figure 5:**
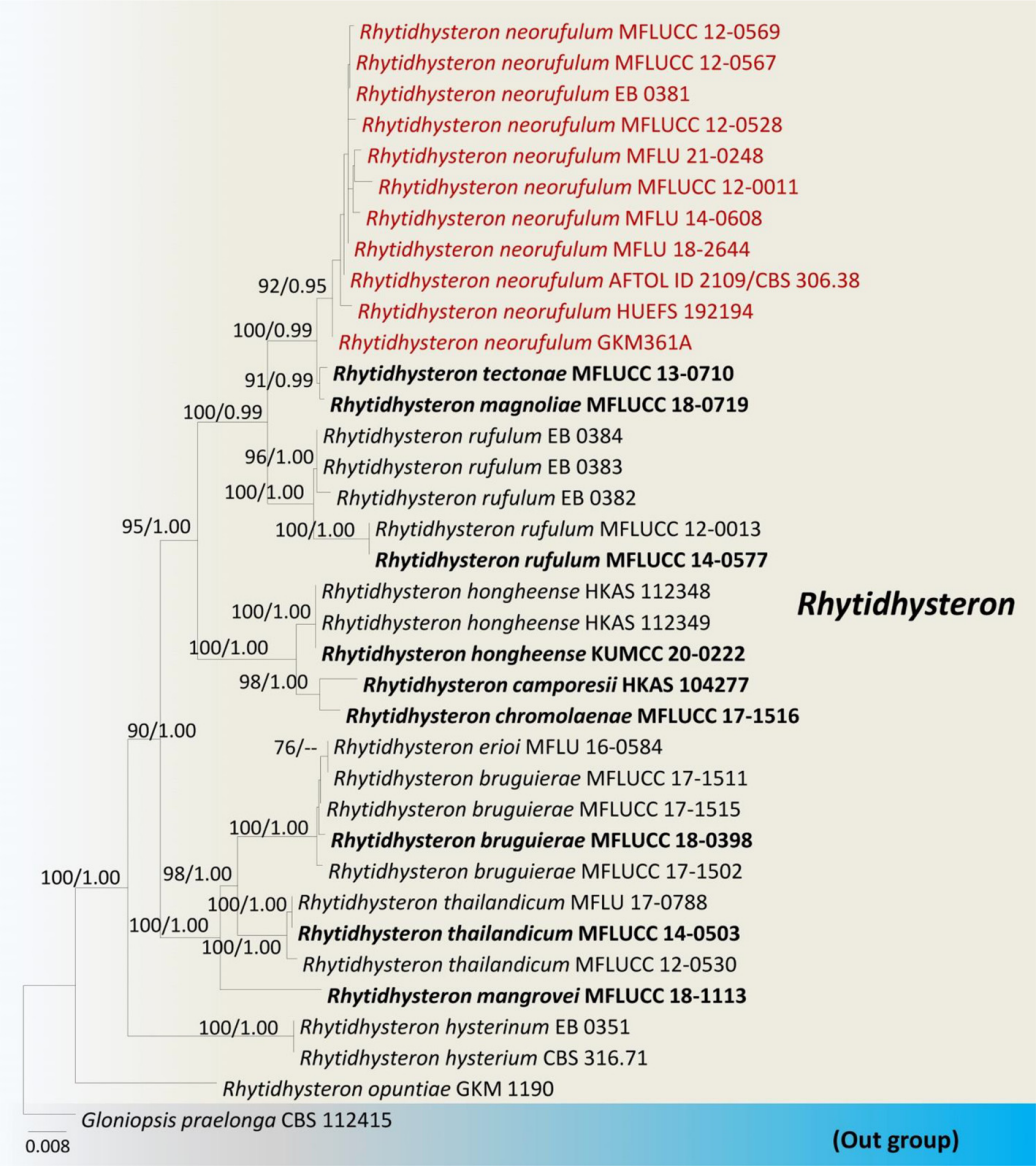
Phylogram generated from maximum likelihood analysis based on combined LSU, SSU, ITS and *tef1* sequence data. The tree is rooted with *Gloniopsis praelonga* (CBS112415). The ex-type strains are in bold, and the *Rhytidhysteron neorufulum* isolates are in red. Bootstrap support values ≥75% from the maximum likelihood (ML) and Bayesian posterior probabilities (BYPP) values ≥0.95 are given above the nodes, respectively.

We reexamined the ascospore characteristics of three isolates of *R. neorufulum* (MFLU 18-2644, MFLU 21-0248 and MFLU 14-0608) and found that they are highly diverse in their shape, color, and septation (**Figure 4**). Ascospores can range from ellipsoidal to fusiform, ellipsoid to oblong or narrowly fusiform, and with pointed or obtuse ends (Thambugala et al., 2016; De Silva et al., 2020). Indeed, a single specimen can possess all these shapes (MFLU 18-2644). Ascospores of *R. neorufulum* have a variety of colors, including hyaline, pale brown, golden brown, and dark brown. The ascospores can be mono-septate, symmetrical, or asymmetrical and constricted at the central septum, while others are 2–3-septate, symmetrical, or asymmetrical and slightly constricted at the central septa. In addition, some ascospores have enlarged second cells from the apex. *Rhytidhysteron neorufulum* has a high phenotypic plasticity of ascospores and ascomata characteristics, and those characteristics may be considered insufficient for delimitation of species of *Rhytidhysteron*. Therefore, species-level delimitation in *Rhytidhysteron* is best accomplished using a multi-gene phylogeny (**Figure 5**) in combination with morphological characteristics.

#### 2.2.2 Morphology variation in *Misturatosphaeria aurantiacinotata*

**Figure 6:**
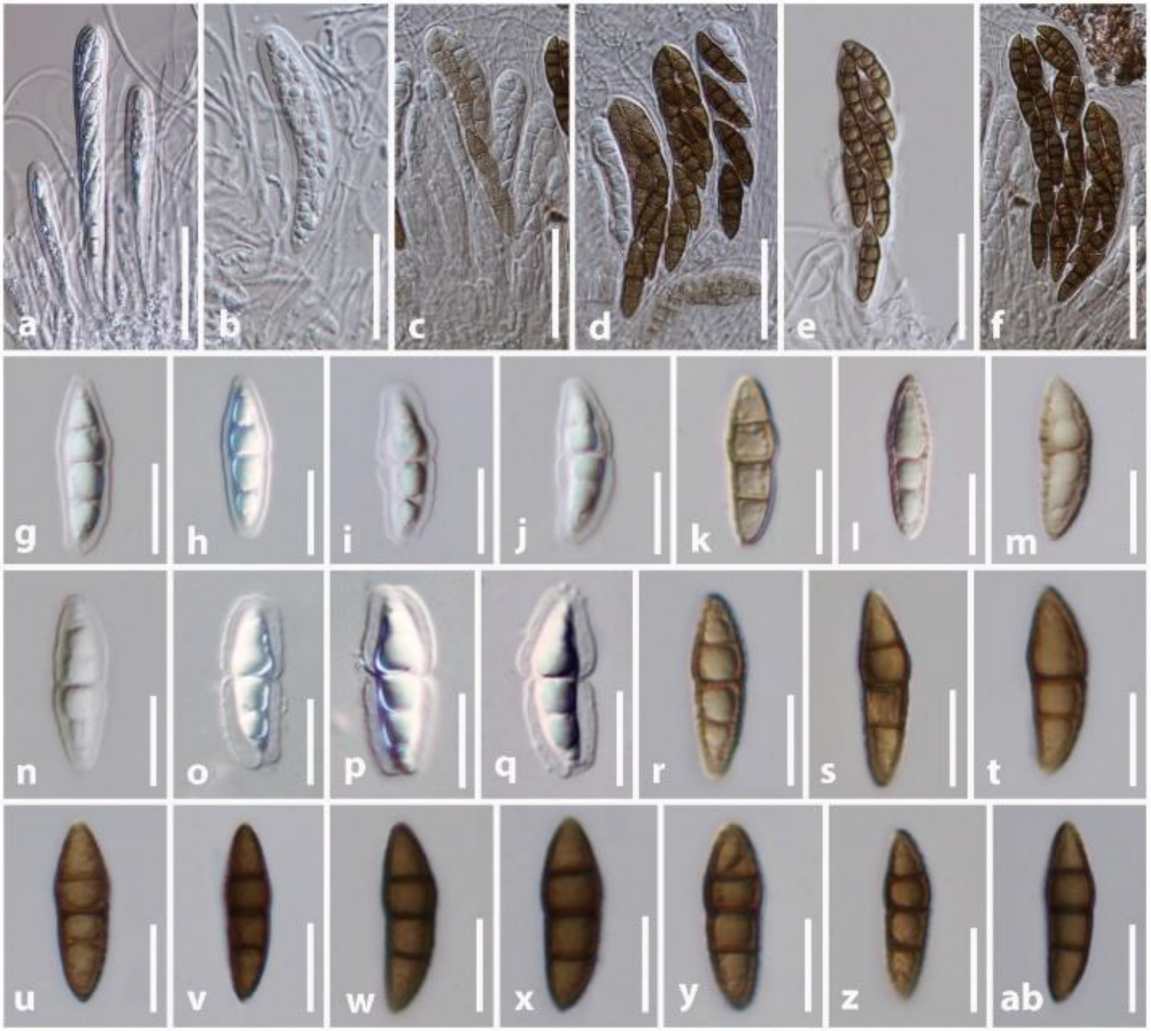
Ascospores variation in *Misturatosphaeria aurantiacinotata* (GKM 1238). **(A–F)** Asci. **(G–AB)** Ascospores. Scale bars: **(A–F)** = 50 μm, **(G–AB)** = 10 μm.

**Figure 7:**
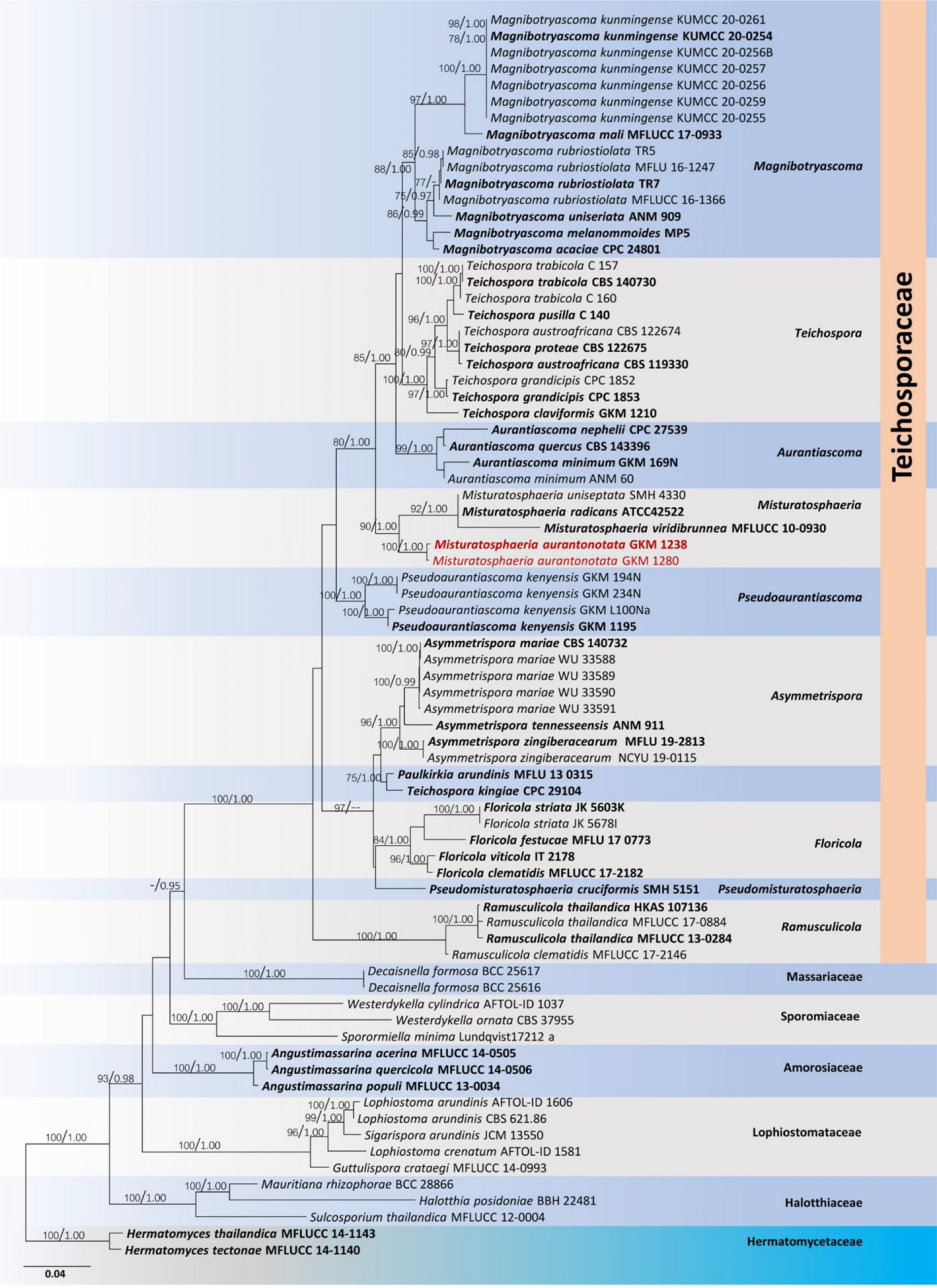
Phylogram generated from a maximum likelihood analysis based on combined LSU, SSU, ITS, *tef1* and *rpb2* sequence data. The tree is rooted with *Hermatomyces tectonae* (MFLUCC 14-1140) and *H. thailandica* (MFLUCC 14-1143). The ex-type strains are in bold, and the *Misturatosphaeria aurantiacinotata* isolates are in red. Bootstrap support values ≥75% from the maximum likelihood (ML) and Bayesian posterior probabilities (BYPP) values ≥0.95 are given above the nodes, respectively.

*Misturatosphaeria* is a member of the family *Teichosporaceae* in the Dothideomycetes. This genus was introduced by Mugambi and Huhndorf (2009) based on mixed ascospore morphological characteristics of this group. It is undeniably true that ascospores of this genus exhibit high phenotypic plasticity even within the same species (GKM 1238). For instance, the ascospore characteristics of *Misturatosphaeria aurantiacinotata* vary from hyaline to light brown or dark brown, are 1–3-septate, deeply or slightly constricted at the central septum and some have a mucilaginous sheath while some lack this feature (**Figure 6**). As such, their ascospores display highly diverse morphological characteristics (Mugambi and Huhndorf, 2009; Thambugala et al., 2015; Tennakoon et al., 2021). Moreover, it is very important to select fruiting bodies of the same level of maturity for morphological examinations, especially with respect to ascospores. Otherwise, there is a possibility of misidentifying a specimen as an entirely different species. In the case of *M. aurantiacinotata*, when only immature ascomata are present on a substrate, it is possible to observe hyaline, 1-septate ascospores that are deeply constricted at the central septum have a distinct mucilaginous sheath. But in mature ascomata, one can observe light brown to dark brown, 3-septate ascospores that lack a mucilaginous sheath (**Figure 6**). Thus, multi-gene phylogeny takes on an important role in the classification of species of *Misturatosphaeria aurantiacinotata* (**Figure 7**).

## 3 Cryptic species - phenotypic noise and taxonomy

A comprehensive taxonomic framework serves as a cornerstone in many biological research endeavors. It is imperative to thoroughly scrutinize and articulate the specific delineation of species within a taxonomic group, as well as to define its boundaries, to establish a robust taxonomic structure. The presence of cryptic species and phenotypic noise often contributes to the lack of taxonomic clarity and leads to confusion within the field (Bochner, 2008; Delić et al., 2017). In some cases, the same species has been listed under different taxonomic names; in other cases, different species have been classified under the same name.

Distinct species may be hidden within species complexes because the characteristics expressed by the phenotype are not sufficient or strong enough to classify them as different species even though they are genotypically different and reproductively isolated. Hence, it causes difficulties in setting up species circumscriptions and delimitations. Furthermore, the same species having several taxonomic names results in an inaccurate taxonomic system, leading to errors in taxonomic applications in other research areas.

There are practical difficulties in detailed observations of microorganisms and even microstructures of both macro- and microorganisms; thus, detecting cryptic species by their micromorphology could be more difficult. However, in the case of pseudo-cryptic species, a detailed re-examination of phenotypic data provides better solutions for correctly identifying cryptic species (Lajus et al., 2015).

In some cases, detailed anatomical characteristics are not supportive enough to get a clear taxonomy. Instead, molecular tools provide better comparisons and more effectively reveal cryptic species (Karanovic and Cooper, 2012; Karanovic et al., 2016). The detailed microanatomical re-description for *Pontohedyle verrucosa* was insufficient to reveal reliable characteristics for diagnosing the two major clades identified within the genus. Thus, the molecular analyses based on four genetic markers (mitochondrial cytochrome c oxidase subunit I, 16S rRNA, nuclear 28S, and 18S rRNA) provided sufficient characterization to discover and formally describe nine cryptic new species (Jörger and Schrödl, 2013).

Moreover, fast-evolving molecular markers are even more effective in revealing cryptic species (Karanovic et al., 2016). For example, the 5´-end of the mitochondrial COI gene has been recommended as the barcoding gene for all species of animals (Hebert et al., 2004). The main benefit of this mtCOI gene is that it has little genetic variation within a species but considerable variation between species (Karanovic et al., 2016). Furthermore, metabarcoding has recently emerged as a prominent tool for species identification. This approach proves particularly valuable when conventional morphological identification of species encounters difficulties, as it hinges on the distinctive genetic information inherent to individual species (Semmouri et al., 2021). Metabarcoding can help distinguish cryptic species by targeting specific barcoding DNA regions (e.g., COI for animals, rbcL for plants, and ITS for fungi). In some cases, phenotypic noise, which includes variation in appearance or phenotype within a single species, can make traditional identification methods less reliable. Metabarcoding can overcome this challenge by focusing on the underlying genetic information, which tends to be more consistent. This helps reduce the impact of phenotypic noise and cryptic species on species identification (De Luca et al., 2021; McInnes et al., 2021; Semmouri et al., 2021). However, when molecular markers are inadequate in clarifying taxonomic uncertainties, whole-genome sequencing emerges as a promising approach to establishing a consistent taxonomy (Thompson et al., 2021; Cai et al., 2023). In bacterial taxonomy, common genetic markers encompass the 16S small subunit ribosomal RNA, a hypervariable region within the genome. Nevertheless, the utility of 16S rRNA genes in *Microcystis* taxonomy has demonstrated their limited informativeness, primarily attributed to the high sequence similarities (>97%) (Harke et al., 2016; Dick et al., 2021). Moreover, while the selected genetic markers may not accurately represent the entire genomic evolutionary landscape, whole-genome sequencing offers a potential solution for establishing a stable taxonomy (Rodriguez-R et al., 2018; Thompson et al., 2021).

In this context, revisiting old taxonomy with modern tools is important to resolve taxonomic confusion. The recent research by Hongsanan et al. (2020) on refining families of the Dothideomycetes expanded information on families in the Dothideomycetidae using new data and molecular tools. Furthermore, the paper provided a refined, updated document on orders and families *incertae sedis* of the Dothideomycetes. When considering phenotypic noise, morphological changes are more readily visible in macroorganisms than microorganisms. Therefore, the morphology of macroorganisms can tell a different story than their genotype. However, physiological characteristics are easily detectable in microorganisms, especially bacteria and viruses. As a result, in many group phenotypes (collectively morphology and physiology) express variations independently from genotypes, which can result in a conflicting taxonomy (Dussex et al., 2018). However, deviations also can be seen in this regard, especially in yeast (Yurkov et al., 2015).

Taxa with large geographic distributions are comprised of cryptic species and phenotypic noise, thus leading to errors in a taxonomic system. For example, many marine taxa have an exceedingly wide geographical distribution (Knowlton, 1993, further discussion in the “Cryptic species–phenotypic noise and biodiversity section”). Hence, marine specimens from widely separated areas are possibly misidentified as a single species but later recognized as a cryptic species complex. Similarly, a huge geographical distribution can lead to phenotypic variations within a single species, resulting in several different species names being applied. Until those different names for the same species are synonymized, probably following molecular phylogenetic analyses, they confuse taxonomic systems.

Integrated taxonomy (taxonomy based on characters from different sources (e.g., from morphology, molecules, ecology or distribution) plays a major in minimizing the effect of cryptic species and phenotypic noise (Padial and De la Riva, 2010; Jörger and Schrödl, 2013). Furthermore, using discriminate characters, based on their quality and suitability in species delineation, instead of adding more and more data is also important in developing a clear taxonomy. Hence, the taxonomic experts of a particular group are there to guide the respective set(s) of characters that will provide the best backbone for the diagnosis of a specific group (Jörger and Schrödl, 2013).

Although cryptic species are phenotypically similar and occur in most groups, those with a well-studied morphology and that contain specimens of a large size show this phenomenon to a lesser extent (Karanovic et al., 2016). Molecular studies or integrated taxonomy of morphology and genetics can resolve this situation and incorporate cryptic species into their component species (Karanovic et al., 2016). For example, four possible cryptic species were found after barcoding 260 North American bird species (Hebert et al., 2004). In contrast, numerous cryptic species were found in subterranean faunas based on molecular analyses with deep morphological examinations (Guzik et al., 2011). In the case of phenotypic noise, molecular phylogenetic tools are essential for the identification of species phylogeny and assigning these to their correct taxonomic ranks.

## 4 Cryptic species - phenotypic noise and evolution

Cryptic species contribute to taxonomic incompleteness, impacting the elucidation of evolutionary events and significantly influencing the delineation and classification of a given taxon. Moreover, these cryptic species obscure the evolutionary trajectories of traits within a specific lineage, thereby complicating species delineation efforts (Karanovic et al., 2016).

Cryptic species are in different evolutionary states. Some cryptic species are in the transition stage from populations to species. They are evolutionarily young, and their morphological descendants have not yet diverged (Delić et al., 2017). Other cryptic species are reproductively isolated from each other, with strong biological barriers between them. These cryptic species are evolutionarily old, and their morphologies provide delimiting characters when examined in detail (Delić et al., 2017). Both of these cryptic species and their evolutionary states are important to develop well resolved evolutionary relationships.

Stochasticity in gene expression causes phenotypic noise (Raser and O’shea, 2005). Within a cell or an individual organism, gene expression can be affected by either extrinsic or intrinsic factors, resulting in gene expression variations (Kaneko and Furusawa, 2008). Intrinsic factors are cell signaling molecules and minor genetic alterations. Examples for those genetic alterations include such things as haploinsufficiency and epistasis (Deutschbauer et al., 2005; Eckardt, 2008; Wang et al., 2014). These genetic alterations are inheritable and cause significant variations in the phenotype. The extrinsic factors are environmental factors. These factors are not significant enough to cause a particular species to separate into different species as described in the species concept (Raser and O’shea, 2005; Eckardt, 2008; Wang et al., 2014); hence it results fluctuations in phenotypes, which are phenotypic noises. These phenotypic noises are at sub-species level of a taxonomically described species. They are actually environmentally sensitive subpopulations of the same genotype. Hence, it is assumed that the actual genotype is being shielded by the external environment, which results in a conflict between genotype and phenotype (de Vienne, 2021). Consequently, correct identification of the distinct phenotypic and genotypic state of a particular species is essential for the understanding of its evolutionary relationships.

Moreover, this is the initial stage of the evolution of isogenic populations with phenotypic noise. This is a deviation from the general evolution process based on the inheritance of beneficial mutations of an organism in a population. Environmentally sensitive subpopulations characterized by phenotypic noise can adapt to a variety of environmental conditions without any genetic base, however with changes in their pattern of gene expression influenced by the environment. This spontaneous adaptation by noise can later be developed to a new environmentally well adapted isogenic population, when the deviation of gene expression level is significant enough to separate them from the main population (Kaneko and Furusawa, 2008).

## 5 Cryptic species - phenotypic noise and ecology

The physiological characters of a species are even more related to ecology. As such, phenotypic noise species are highly influenced by their environment. Although cryptic species are phenotypically undifferentiated, their ecology provides more possibilities for their discovery. As an example, Peintner et al. (2019) examined the use of ecological factors in resolving cryptic species in the fungal genus *Fomes*, and they concluded that volatile organic compounds serve a better outcome in species delimitation and discovering cryptic species in the future (Peintner et al., 2019). In addition, Bickford et al. (2007) mentioned that cryptic species are more common in insects and reptiles, and they tend to occur more in the tropics than temperate regions.

Cryptic species are common and can be found in any kingdom of the Tree of Life (McGuire, 2018). However, cryptic plant species are comparatively unclear and rare because genetic and molecular tools used to identify cryptic species in other organisms do not really fit with plants (Shneyer and Kotseruba, 2015). Furthermore, more records of cryptic plant species can be determined with more detailed examination of plants and with the improvement of new tools such as nuclear ribosomal DNA sequencing (Okuyama and Kato, 2009; Shneyer and Kotseruba, 2015). Okuyama and Kato (2009) examined the Asian endemic perennial lineage of *Mitella* (*Asimitellaria*; Saxifragaceae) based on nuclear ribosomal DNA sequences and discovered ten distinct biological species, including two new species. A multidisciplinary study, which combined cytogenetic analyses and phylogenetic analyses on two plastid and five nuclear genes, revealed the taxonomic separation of three distinct species in the *Brachypodium distachyon* complex, a model grass for cereals (Catalán et al., 2012). Moreover, cryptic species in plants can be differentiated by the secondary metabolites they produce. These secondary metabolites associated with pest and disease susceptibility, stress tolerance, interactions with soil microbes, attraction of pollinators, and palatability to herbivores or simply the ecological niche of a particular plant species (Shneyer and Kotseruba, 2015).

Gene expression variations associated with non-genetic factors are closely connected with ecology. Hence, phenotypic noise species are adaptive subpopulations to a particular habitat. The long-term ecological influences on those subpopulations or specific phenotypic noise also increase the evolution rate of the population. Moreover, those specific phenotypic noise species bear acquired characters, which can be referred to as adaptations to their environments. In multicellular organisms, acquired characters would be in germplasm (genotype) or somatic cells (phenotype) (de Vienne, 2021). Somatic cell DNA mutations can impact individuals but not their progeny (Hurle, 2022). However, recent agricultural advances in tissue culture techniques and somatic cell genetics allow the regeneration of plants from cells in culture. In somatic cell genetics, even the changes in somatic cells can be transferred to the next generation (NCBI, 2022).

Agricultural research and ecology-based applications are more affected by cryptic species. One of the best examples is the development of biocontrol agents. The success of a biocontrol agent is based on a solid understanding of the biocontrol agent and the target organism, the details of their life cycle, preferred habitats, and foods (Walter, 2003; McGuire, 2018). The presence of cryptic species within either the pest population or the biocontrol agent population can fail the control method for no apparent reason (Walter, 2003; McGuire, 2018). Distinct species react differently to control methods. Correct identification and a better understanding of host and pathogen species are very important in developing control strategies (Walter, 2003; McGuire, 2018). This applies not only to biological control but also to any of the pathogen control methods affected by cryptic species.

The ecological niche of the organism is very important in discovering cryptic species within pathogenic taxa. More studies on pathogen-host-environment interactions are required to increase the understanding of the ecology of cryptic species (Jung et al., 2017). Also, there is a higher possibility of misleading species identification by phenotypic noise. As the plant host plays a major role in speciation, the physiological characters of the pathogenic organism have also turned out to be species-specific (Peintner et al., 2019). Hence, the same species can have different phenotypes within different hosts. Conversely, host interactions can use as a delimiting character to resolve cryptic species. An example for the host and cryptic species relationship showed up in an analysis of anther smuts (Genus *Antherospora*) on *Muscari* spp. The molecular phylogeny revealed three distinct lineages that were correlated with host plants with slight morphological differences. These lineages were assigned to three cryptic species: *Antherospora hortensis* sp. nov. on *Muscari armeniacum, A. muscari-botryoidis* comb. nov. (syn. *Ustilago muscari-botryoidis*) on *M. botryoides*, and *A. vaillantii s. str*. on *M. comosum* and *M. tenuiflorum* (Piątek et al., 2013). Furthermore, new cryptic species of *Teratosphaeria*, which is a serious pathogen of *Eucalyptus* were discovered from the phylogenetic analyses of gene regions of Internal transcribed spacer (ITS), β-tubulin (tub2), and translation elongation factor (tef1) (Andjic et al., 2016). Crespo and Lumbsch (2010) also examined cryptic species on lichens.

Furthermore, species complexes such as *Microcystis* are ecologically much bounded. The taxa within complexes vary across the environmental and seasonal conditions (Frangeul et al., 2008; Cai et al., 2023). Hence, the phenotypic characters become useless in species delimitation, and molecular tools provide promising results. However, the high genome variability of *Microcystis* due to high genome plasticity and horizontal gene transfer causes the use of genetic markers to be inadequate for species delimitation. In such cases, advanced molecular tools such as pangenomics reveal cryptic diversity within those complexes (Cai et al., 2023).

## 6 Cryptic species - phenotypic noise and biodiversity

The “species concept” or “species level” concept plays a fundamental role in biodiversity (Lefébure et al., 2006). Estimating species numbers is an important criterion for both fundamental and applied biodiversity perspectives. Determining the boundaries of species is essential for species number and diversity estimations (Sites and Marshall, 2003). Although many species boundaries exist, their delimitation has no clear and operational criterion (Lefébure et al., 2006). The presence of cryptic species or failures in detecting existing species results in an underestimation of true levels of biodiversity (Lefébure et al., 2006; Padial et al., 2010). Conversely, the same species can be identified by different names as it has many phenotypic noise species, which falsely increases the number of species.

Moreover, biodiversity conservation has also been disturbed by cryptic species. This is because there are species hidden within cryptic species complexes, and these are often rare, have low populations, are seriously threatened, and thus are highly endangered and require urgent conservation efforts. However, conservation programs for undiscovered and unavailable species cannot be carried out. In addition, the formal naming of cryptic species is essential to enable them to be added to conservation policies and faunal listings (Delić et al., 2017). Recent research on the South European cryptic complex of the subterranean amphipod *Niphargus stygius sensu lato* used uni- and multilocus delineation methods. Molecular analyses showed that the newly discovered species came from several different subgroups within the genus, and that these subgroups coexist while showing no signs of lineage sharing. Those newly discovered cryptic species have increased the number of subterranean endemics in Slovenia by 12 and in Croatia by four previously underestimated species. Moreover, the new taxonomic additions renewed the national Red Lists, as it previously included mostly species with large ranges but omitted critically endangered single-site endemics (Delić et al., 2017).

True cryptic species within a species complex cause huge errors in biodiversity analyses, as discussed above. However, the morphological analyses have considerable limitations in discriminating species and need to be more robust for describing biodiversity at the species level. Therefore, nonmorphological techniques such as genetic analyses and investigations of behavioral, physiological, and other traits must be employed (Lajus et al., 2015). Furthermore, as some studies have included thorough morphological and molecular examinations to detect cryptic species, they do not lead to species descriptions (Pante et al., 2015). This results in an inflation of alpha diversity estimation (Chessman et al., 2007; Morrison et al., 2009; Karanovic et al., 2016). In this context, the phylogenetic species concept is essential in recognizing a far greater number of much less inclusive units. Moreover, the phylogenetic species concept closely examines the same group of organisms, resulting in different species identities, species ranges, and number of individuals (Agapow et al., 2004). Literature reviewing and analyzing organisms that were categorized under phylogenetic and nonphylogenetic concepts show a significant difference, in analyses based on a phylogenetic species concept showing 48% more species than nonphylogenetic concept analyses (Agapow et al., 2004).

The traditional molecular techniques faced challenges when dealing with extensive sample analyses, which often hindered conducting large-scale biodiversity assessments. As a result, researchers outside the taxonomy field have adopted metabarcoding tools for specimen identification. Metabarcoding also holds promise for tasks such as the detection of new species, the revelation of cryptic species, the identification of non-native or invasive species, and the assessment of taxonomically meaningful variations within species that have broad or geographically scattered distributions (De Luca et al., 2021; McInnes et al., 2021; Semmouri et al., 2021). Marine protists have been perceived as having low diversity and a widespread distribution. However, recent research has revealed that many protist species are, in fact, intricate assemblages of cryptic species, often confined to specific biogeographic regions. Nevertheless, the detection of these cryptic species is frequently hindered by limitations in sampling coverage and the application of methods, such as phylogenetic trees, which are less suited for identifying relatively recent divergence and ongoing gene flow (De Luca et al., 2021). In such scenarios, metabarcoding emerges as an ideal solution. De Luca et al. (2021), successfully unraveled the complexities within the *Chaetoceros curvisetus* (Bacillariophyta) species complex by employing two complementary metabarcoding datasets. Species are diverse and common, especially in marine habitats (Knowlton, 1993). *In situ* observation of marine taxa is difficult. Furthermore, many marine invertebrate taxa require *in situ* detailed observations of live specimens by experts or well preserved and fixed specimens to be properly identified, conditions that are hard to fulfill, especially when it comes to large-scale marine inventories. Moreover, since it is possible to spread larvae/adults in a homogenous sea by ocean currents, marine organisms have a wider geographical distribution than terrestrial ones (Knowlton, 1993; Sundberg et al., 2009). This huge distribution of marine organisms results in errors in identifications and later gives rise to cryptic species complexes. Misidentification of the same species based on its phenotypic noise is even possible. Some taxa are affected by these factors more than others, such as small, soft-bodied, marine invertebrates with few morphological characters useful for taxonomy (Knowlton, 1993; Sundberg et al., 2009). Furthermore, certain organisms remain concealed within vast ecosystems because of their unculturable nature and significantly smaller population sizes (He et al., 2015; Ryberg, 2015). However, studies on fungi inhabiting unique environments have revealed that numerous fungi previously considered ‘unculturable’ can be cultivated on specific substrates and under particular conditions. Additionally, high-throughput amplicon sequencing, shotgun metagenomics, single-cell genomics, and Molecular Operational Taxonomic Units (MOTUs) serve as a valuable means to unveil these hidden organisms and offer a promising tool for delineating species boundaries and quantifying biodiversity (He et al., 2015; Ryberg, 2015; Wu et al., 2019).

## 7 Discussion

This paper discusses the two major concepts, cryptic species and phenotypic noise, which affect species concepts. The species concept is the basis of taxonomy, evolution, ecology, and biodiversity. As the kingdom of fungi has made many contributions to biotechnology and applied sciences, this review is expected to improve the identification of fungal species and aid in developing future fungal research applications.

Several branches of species concept are currently used, *viz.* morphological species concept, biological species concept, evolutionary species concept, and phylogenetic species concept (Taylor et al., 2000). A better understanding of each concept is essential for investigations on cryptic species and phenotypic noise concepts. Cronquist (1978) adopted the morphological species concept and refers to a community with distinctive morphological characters sufficient to entitle them to a specific name (Aldhebiani, 2018).

The biological species concept applies to a group with a distinct ecological niche that is reproductively isolated (Mayr, 1982), while an evolutionary species refers to a single lineage of ancestor**-**descendant populations with a unique evolutionary history (Simpson, 1961; Wiley, 1981; Paris et al., 1989). The phylogenetic species concept is outside of cladistic analysis and focuses on possible evolutionary processes contributing to species formation, including biotic and abiotic (even random) factors (Wheeler, 1999). A new concept being proposed is the modern species concept, which is focused on the relationship (phenetic or phylogenetic) between individuals (Aldhebiani, 2018).

However, there is no clear congruence between intraspecific variations and species boundaries since some taxonomic studies and many identifications are still based on morphological characters. This simply results in cryptic species, which are phenotypically similar but genotypically distinct, as well as phenotypic noise from species that are phenotypically distinct but genotypically identical. Hence, the cryptic species represent genetic variations of a population. Non-genetic variations are phenotypic variations that arise from different expressions of a genotype. Phenotypic variations are persuaded by varying environmental cues and categorized into two types: phenotypic plasticity and developmental noise (Saito et al., 2023). Phenotypic plasticity is a common act of a biological population or among individuals of a single species as a response to their recent environment by changing their morphology, physiology, or behavior. This is the foundation of how organisms learn, interact with their environment, and adapt to their environment during their lifetime (Matthey-Doret et al., 2020). Therefore, phenotypic plasticity refers to expressing traits that depend on the environment. However, the traits that change unpredictably in different environments are usually said to be ‘noisy’ rather than plastic (Gomulkiewicz and Stinchcombe, 2022). Hence, in this article, we are addressing the concept of phenotypic plasticity using the term phenotypic noises.

Nowadays, DNA sequence information has become the only truly reliable factor for species identification. However, morphological data still play a crucial role in taxonomy, biodiversity, and ecological studies as these data are the primary key to identifying a species, and sometimes there are practical difficulties in obtaining molecular data. Moreover, the concept of phenotypic noise can also result in the occurrence of different individuals or groups of individuals within an isogenic population (Engl, 2019). Hence, using a combination of molecular phylogenetic approaches and morphological and physiological data make a fine-scale taxonomy possible (Jörger and Schrödl, 2013). Moreover, once cryptic species are discovered as a result of the availability of molecular data, there must then be a detailed examination and well documented description of newly discovered taxa using visible external or internal differences to avoid misidentifications in the future.

Most traditional taxonomic studies for some fungal groups, especially those that are unable or difficult to grow on artificial growth media, such as lichenized fungi and discomycetes, are still based on morphological characteristics. Hence, this results in classifying different species under the same taxonomic name. Later, these can be recognized as cryptic species, which causes a major conflict in natural classification systems. Furthermore, setting up evolutionary relationships is difficult with cryptic species. Generally, for most organisms, genetic changes tend to happen slowly than morphological changes (Hanken and Carl, 1996; Carroll, 2005; Hall and Strickberger, 2008). However, when genetic changes happen more rapidly than morphological changes, we see only the phylogenetic differences whereas the morphological changes are not yet discernible. The concept of phenotypic noise results in morphologically and physiologically diverse individuals within isogenic populations (Engl, 2019). Furthermore, Fungi have a unique mode of growth that allows them to adapt quickly to changing environments. Unlike other multicellular organisms (animals and plants), fungi evolved complex multicellularity through filamentous intermediate stages (Nagy et al., 2017). Hence they can exhibit a wide range of phenotypic or functional plasticity in response to environmental cues (Nagy et al., 2017). These changes can occur rapidly and do not always require genetic changes (Naranjo-Ortiz and Gabaldón, 2019). Moreover, since genetic changes can contribute to morphological changes in fungi, it is not accurate to say that genetic changes happen more rapidly than morphological changes in this group of organisms. The relationship between genetic and morphological changes in fungi is complex and varies depending on the specific context (Naranjo-Ortiz and Gabaldón, 2019). This would also result in confusion not only in taxonomy and evolution but also in biodiversity and ecology (Korshunova et al., 2019).

Here, we provide three fungal examples to understand the cryptic species concept and phenotypic noises concept clearly. Around 2–11 million estimated species and around 3 million predicted species are in kingdom fungi, and only around 150,000, a tiny fraction of the total estimation, have been formally described (Phukhamsakda et al., 2022). Compared to plants and animals, fungi have simple body plans that are often morphologically and ecologically concealed and continuously challenging for precise identifications and species delimitations (Lücking et al., 2020). Plant pathogenic fungi cover one of the most significant fractions of total estimated fungi and play an important ecological and economic role; hence, they are directly involved with the development of effective food security, crop protection, and pest risk assessments (Cai et al., 2011; Li et al., 2020). For example, rust and smut fungi collectively cover 10% of all known fungi. Therefore, DNA sequence data have recently been extensively employed in developing stable taxonomy for plant pathogenic fungi (Cai et al., 2011). The next crucial ecological group of fungi is saprobes for the earth’s nutrient cycling. The examples provided here are saprobes, which evolve fast with environmental stimuli (Naranjo-Ortiz and Gabaldón, 2019). This causes fast-evolving species concepts and delimitations. Hence, the fungi kingdom needs extensive analyses and comprehensive species identification methods. Recently, a conceptual framework was analyzed and suggested for identifying fungi (Lücking et al., 2020). Further, the approach encourages the integrative (polyphasic) taxonomy for species delimitation, which combines phylogeny, phenotype, and reproductive biology. This facilitates evaluating a wide range of diagnostic characteristics, either phenotypic, molecular, or both (Lücking et al., 2020). However, polyphasic approaches are being limited by the availability of data and analyzing capacity of particular groups, while it is well suited for some groups. The effectiveness of polyphasic approaches for identifying yeast as a case study was widely investigated (Yurkov et al., 2015; Boekhout et al., 2021). The advantage of polyphasic approaches over traditional morphological character-based approaches is that species ultimately depend on species recognition approaches and the number of characters in traditional methods. Considering the dichotomous key-like approach, species recognition is based on a few characteristics, such as categorical (spore types, color) or numerical range variables (spore size). In polyphasic approaches, taxonomy based on many characteristics [e.g., assimilation tests for yeasts (Yurkov et al., 2015; Boekhout et al., 2021)] and results in an in-depth understanding of the phenotype and/or niche concepts which aid to recognize cryptic species and phenotypic noises and minimize the confliction in stable systematics.

Phylogeny-based clade-specific evolutionary histories are mostly used to verify species identifications, a pivotal step in taxonomy. However, no single tool for identifying fungi and internal transcribed spacer (ITS) is still considered the first diagnosis, particularly in metabarcoding studies. Secondary DNA barcodes are long-established for resolving taxonomy and for precise identifications where they do not provide sufficient distinctness (Lücking et al., 2020). When considering the molecular markers for resolving cryptic species, it is important to know that identifying “what we call cryptic” depends on the markers used. Therefore, it is more important to ensure congruence between the different markers. Simply adding more and more increasingly variable gene loci can identify more lineages in a population, but the lineages may not necessarily represent species. This problem of applying species names to lineages within a species is becoming more common in the literature with respect to the diversity of fungi lineages being described as species. These pitfalls can be resolved using single-copy orthologs genes and coalescence-based methods (Shen et al., 2016; Maharachchikumbura et al., 2021). Single-copy orthologs are genes that are present only once in the genome of each species, and have descended from a single ancestral gene present in their last common ancestor (Aguileta et al., 2008; Ren et al., 2016; Shen et al., 2016; Maharachchikumbura et al., 2021). Applications of whole genome sequencing are becoming increasingly common in fungal studies, as complete genomes provide in-depth knowledge and comparisons on the evolvability of the genomes and, consequently, reveal the species’ true ancestry. Furthermore, using complete genome information is more beneficial than drafting genomes, as it provides a better understanding of species and their genome and genetic dynamics (Boekhout et al., 2021). The species recognition in some groups of fungi is tightly connected to species concepts of their hosts and vectors. This is especially important for parasitic fungi, depending on their host species. Some plant-pathogenic fungi have a broad host range, while the fungal family *Erysiphaceae* causes considerable damage to many crop plants, including Grapevine, *Eucalyptus*, Rubber, cucumber, tomato, onion, pepper and potato (Ekanayaka et al., 2019), some are highly limited in the range of plant species [e.g. Fungal family *Cyttariaceae* is host specific on *Nothofagus* spp (Ekanayaka et al., 2019)] or even cultivars that they cause diseases (strict host-parasite association) (Li et al., 2020). Several recent taxonomic studies of fungi include the host of fungi collected into their descriptions, and the host used as a character in species recognition (Hongsanan et al., 2020; Hyde et al., 2020), which would be helpful in accurate species identification. Furthermore, Cryptic sibling species can be specialized in different ecological conditions and often become host-specific. For instance, the fungus *Microbotryum violaceum* causes another smut disease in Caryophyllaceae plants, and the degree of specialization and gene flow between strains on different hosts are becoming controversial. Molecular phylogenetic analyses using single-copy nuclear genes help resolve taxonomic confusion in *M. violaceum* from 23 host species and different geographic origins (Le Gac et al., 2007). Hence, molecular phylogenetic analyses play a crucial role in identifying cryptic species. Moreover, host specificity indexes, such as S_TD_, which measures the average taxonomic distinctness among the host species used by a parasite, weighted for the parasite’s prevalence in the different hosts (Poulin and Mouillot, 2005) and specific analyses such as Global and individual ParaFit tests to construct a plant–pathogen evolutionary association network (Zeng et al., 2019; Chen et al., 2022) can be applied to analyze to investigate host and fungal relationship and their coevolution. This also provides a broad spectrum of data on those fungal groups, specifically in niche concepts.

Correct fungal species identification is vital as the kingdom of fungi plays a crucial role in applied sciences. For instance, disease diagnosis and medical prescriptions are based on correct species identifications in the pharmaceutical industry and medical sciences. Cryptic *Aspergillus* species cause cryptic aspergillosis and are becoming more prevalent in humans and can cause significant morbidity and mortality in immunocompromised individuals. Moreover, cryptic *Aspegillus* species are causing invasive diseases and are usually more resistant to common antifungal therapies. Therefore, the correct identification and characterization of these fungi have both epidemiological and clinical implications when evaluating the impact of such species in aspergillosis (Fernandez-Pittol et al., 2022; Ninan et al., 2022). Furthermore, accurate species identification can reveal important information regarding possible biochemical properties of a particular species, which can be explicitly applied to drug discoveries (Raja et al., 2017).

Cryptic species represent a portion of the missing fraction of biodiversity (Vodă et al., 2015). Consequently, resolving cryptic species is important as it reveals the true level of biodiversity and thus contributes to conservation efforts. Some cryptic species may be seriously threatened but unavailable for conservation programs as they remain undescribed (Delić et al., 2017). The evolutionary state of the cryptic species (See cryptic species and evolution) is important with respect to conservation programs. However, the biological properties and their relationship to the recent ancestors relevant for the conservation of cryptic species (i.e., sexual reproduction, spore germination, etc.) are often not known (Delić et al., 2017). Inappropriate management of endangered species can result in these being more threatened than previously thought (Niemiller et al., 2013; Brodersen and Seehausen, 2014; Delić et al., 2017).

During the analyses of highly diverse species, mislabeling and insufficient investigations lead to identifying the same species in different names because of phenotypic plasticity. Later, this can be corrected in taxonomic revisions, and the misidentifications are synonymized under the correct names. However, having many synonyms for a single species still needs to be more accurate. In this regard, conducting taxonomic revisions [e.g (Cruz-Morales et al., 2019; El-Banhawy et al., 2021)] and taxonomic outlines [e.g (Wijayawardene et al., 2020)] that provide recent taxonomic updates on taxa at one place is very important and helpful for precise species identifications.

Resolving cryptic species complexes and identifying phenotypic plasticity of a species are highly demanding but critically important in taxonomy and taxonomic applications in other areas such as evolution, ecology, and biodiversity. It provides a better understanding of species, their distribution patterns in the Tree of Life, and the underlying story of their evolution. We hope this review will provide an overall understanding of cryptic species, phenotypic noise, and their effects. Also, this should encourage more detailed analyses of morphological characters with the combination of molecular phylogenetic analyses, which can provide fine scale taxonomy.

## 8 Conclusions

“Cryptic species” refers to a situation in which two or more species are morphologically indistinguishable in their original descriptions and have been erroneously classified under the same scientific name. “Phenotypic noise” refers to different phenotypic states of the same isogenic population. Both are contributing to evolution but in two opposite ways.

Cryptic species and phenotypic noise create confusion in taxonomic structure and leave huge gaps in describing the flow of the evolutionary process. Molecular tools are the only way to detect them easily. However, a detailed examination of macro-, micro-, external, and internal morphological characters also provides a better endorsement of taxonomic status. Both concepts hide the real biodiversity and inhibit a clear understanding of species evolution and their ecological significance.

Although members of a particular species may appear to be similar, several to many distinct species can be present. In other words, many unrecognized species may be hidden as cryptic species. Many important taxa may need to be protected but are already extinct within cryptic species complexes. Conversely, species limitations must be well identified as the same species could be differently identified based on its phenotypic plasticity for different environmental conditions. Cryptic species and phenotypic variations of the same species also affect studies of ecology and agricultural research, especially in relation to plant pathology and developing biocontrol agents, where these concepts can pose significant obstacles.

Cryptic species result from the species evolution process. Discovering cryptic species and identifying the phenotypic noise of particular species helps develop a fine-scale taxonomic system and improve its applications in areas such as evolution, ecology, and biodiversity.
